# Supplementary material for: Motif-Targeting Phosphoproteome Analysis of Cancer Cells for Profiling Kinase Inhibitors
Source: Cancers (Basel). 2022 Dec 23;15(1):78. doi: 10.3390/cancers15010078 (PMC9817674; doi:10.3390/cancers15010078)
Supplement: Supplementary file 1 [file cancers-15-00078-s001.zip › cancers-2048382-supplementary.pdf]

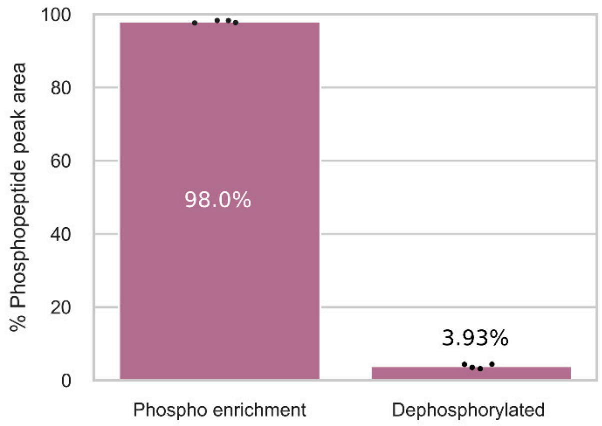


**Supplementary Figure S1.** Efficiency of phosphopeptide enrichment and subsequent dephosphorylation. The sum of the identified phosphopeptide peak areas is divided by the sum of all identified peptide peak areas. Phospho enrichment: The results from phosphoproteome analysis after phosphopeptide enrichment. Dephosphorylated: The results from the one after phosphopeptide enrichment and subsequent dephosphorylation.

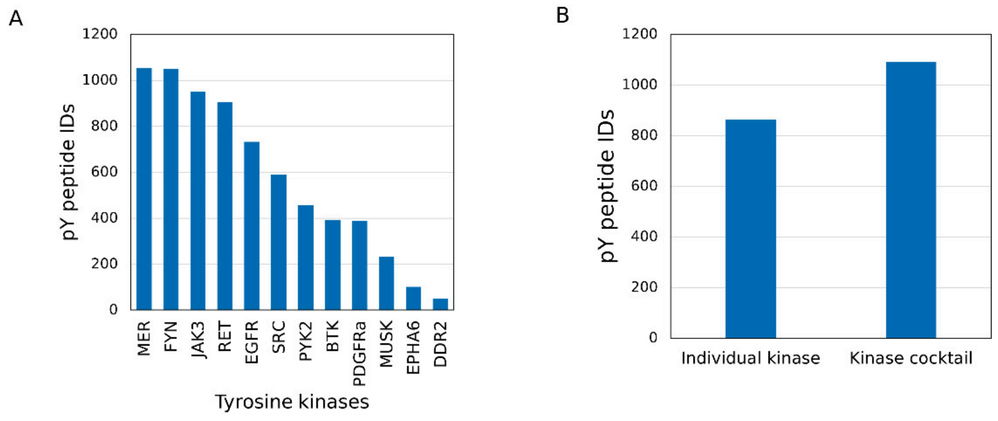


**Supplementary Figure S2**. Tyrosine kinase screening with the motif-targeting workflow. To find the best combination of kinases, 12 tyrosine kinases were screened. All of the samples here were analyzed by LC/MS/MS with the TripleTOF5600. (A) The identification number of phosphopeptides for each kinase. (B) The identification number in triplicate analysis of the samples treated with each of the kinase (FYN, JAK3, and MER) individually (Individual kinase) and after treatment with a cocktail of all three kinases (Kinase cocktail).

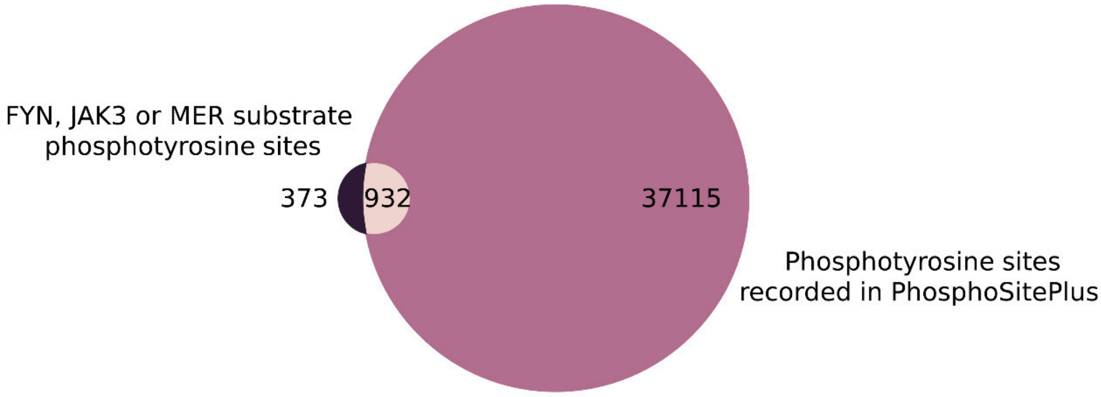


**Supplementary Figure S3.** Comparison of phosphotyrosine sites identified in motif-targeting workflow with public PhosphoSitePlus (www.phosphosite.org) database. The venn diagrams showed the overlap between two data sets. PhosphoSitePlus phosphosite information was extracted from the Phosphorylation_site_dataset file (Nov 18 2022).

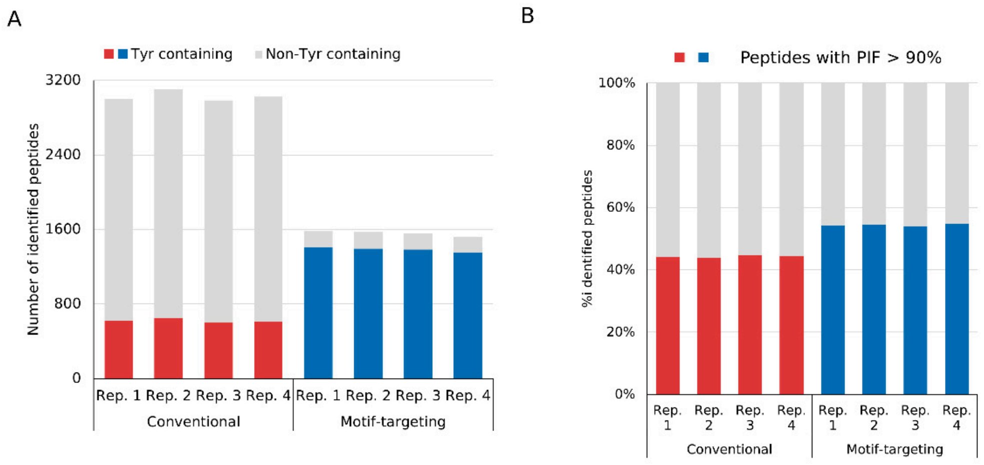


**Supplementary Figure S4.** Reproducibility of the results obtained by motif-targeting workflow. (A) The bar chart shows the identified number of peptides in each experimental workflow. The peptides are categorized based on the presence of tyrosine residues. (B) The bar chart shows the ratios of high-purity matches (PIF>90%) among the peptides identified by the conventional and motif-targeting workflows.

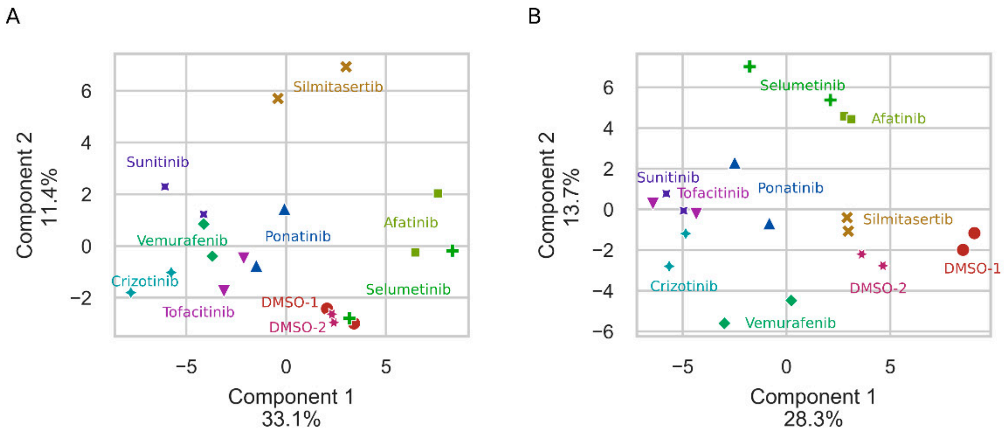


**Supplementary Figure S5.** Principal component analysis of the inhibitor-treated HeLa cells. The quantitative results obtained using (A) the conventional workflow and (B) the motif-targeting workflow were used for the analysis.
